# Isolation of *Burkholderia pseudomallei* from a goat in New Caledonia: implications for animal and human health monitoring and serological tool comparison

**DOI:** 10.1186/s12917-024-03957-5

**Published:** 2024-03-23

**Authors:** Anais Desoutter, Thomas Deshayes, Fabien Vorimore, Bernice Klotoe, Benoit Durand, Julien Colot, Gabriel Wagner-Lichtenegger, Ivo Steinmetz, Apichai Tuanyok, Karine Laroucau

**Affiliations:** 1 LNC (Laboratory of New Caledonia), Animal Health Department, Paita, New Caledonia; 2 Anses, Animal Health Laboratory, Bacterial Zoonosis Unit, Maisons-Alfort, France; 3 Anses, Identypath, Maisons-Alfort, France; 4 Anses, Animal Health Laboratory, Epidemiological Unit, Maisons-Alfort, France; 5 Territorial Hospital Center Gaston-Bourret, Medical Biology Laboratory, Noumea, New Caledonia; 6 Diagnostic and Research Institute of Hygiene, Microbiology and Environmental Medicine, Medical University of Graz, Graz, Austria; 7 Department of Infectious Diseases and Immunology, College of Veterinary Medicine, and Emerging Pathogens Institute, University of Florida, Gainesville, FL 32608 USA

**Keywords:** *Burkholderia pseudomallei*, New Caledonia, Goat, Serology, ELISA, Luminex

## Abstract

**Background:**

Melioidosis is a serious bacterial infection caused by *Burkholderia pseudomallei*, a gram-negative bacterium commonly found in soil and water. It can affect both humans and animals, and is endemic in regions such as Southeast Asia and Northern Australia. In recent years, there have been reports of an emergence of human melioidosis in other areas, including New Caledonia.

**Results:**

During standard laboratory analysis in New Caledonia in 2021, a strain of *B. pseudomallei* was isolated from a goat. The strain was characterized using both MLST and WGS techniques and was found to cluster with previously described local human strains from the area. In parallel, several serological tests (CFT, ELISA, Luminex (Hcp1, GroEL, BPSS1840), arrays assay and a latex agglutination test) were performed on animals from the farm where the goat originated, and/or from three other neighboring farms. Using two commercial ELISA kits, seropositive animals were found only on the farm where the infected goat originated and tests based on recombinant proteins confirmed the usefulness of the Hcp1 protein for the diagnosis of melioidosis in animals.

**Conclusions:**

Despite the regular reports of human cases, this is the first confirmed case of melioidosis in an animal in New Caledonia. These results confirm the presence of the bacterium in the region and highlight the importance of vigilance for both animal and human health. It is critical that all health partners, including breeders, veterinarians, and biologists, work together to monitor and prevent the spread of the disease.

**Supplementary Information:**

The online version contains supplementary material available at 10.1186/s12917-024-03957-5.

## Background

Melioidosis is an infectious disease caused by *Burkholderia (B.) pseudomallei*, a bacterium commonly found in soil and water in endemic areas such as Southeast Asia and Northern Australia [1, 2]. The presence of *B. pseudomallei* in soil is strongly influenced by climatic events, with higher numbers of melioidosis cases typically observed during the rainy season [3]. This pathogen is a member of the *B. pseudomallei* complex, which includes several species such as *B. pseudomallei*, *B. mallei,* and *B. thailandensis* [4]. Of these, *B. mallei*, which evolved from *B. pseudomallei*, is responsible for glanders [5]. *B. mallei* differs from *B. pseudomallei* in its inability to survive in environmental conditions and has a more limited host range, affecting mainly equids [6].

Recent global estimates from 2015 suggest that there are approximately 165,000 human cases of melioidosis annually, highlighting its potential global spread, including to the Pacific, Africa, and the Americas, where human and occasionally animal cases are increasingly being reported along with environmental evidence of the bacteria [4, 7–11]. The importance of melioidosis lies in its ability to cause severe disease in both humans and animals through environmental transmission routes, including inhalation, ingestion, or skin contact.

In humans, this infection presents with a spectrum of symptoms ranging from mild, localized infections to life-threatening systemic disease that can lead to sepsis and death. The severity and the susceptibility of the disease is closely related to the immune status of the host, with increased susceptibility observed in immunocompromised individuals, including those with diabetes mellitus and chronic alcohol abuse [1]. Relapses after many years and chronic infections are also described [1]. Diagnosis of melioidosis is often difficult due to its non-specific symptoms and diverse clinical manifestations [2]. The most reliable method of diagnosis is to culture the bacterium from clinical samples such as blood, urine, or sputum. However, it can take several days to obtain a positive result and there is a risk of false-negative results. Alternative diagnostic techniques include serology, molecular techniques such as PCR, and imaging studies such as CT scans [2, 12, 13].

In animals, melioidosis has been reported in a variety of species, including dogs, cats, horses, goats, and cattle. The environmental factors responsible for contamination and disease in animals are often identical to those that affect humans, sometimes resulting in high rates of morbidity and mortality [6]. Serological tests for melioidosis in animals, such as IHA, ELISA and ICT, are mostly found in research laboratories because there are no commercial tests or control sera available for all susceptible animals [14].

While human cases of melioidosis have been frequently reported in New Caledonia since 1999 [15, 16], no confirmed animal cases have yet been identified. However, a serological study conducted in the 1980s on various animal species had revealed seropositive animals, indicating potential exposure to the bacteria.

In this short communication, we present the first confirmed animal case of melioidosis in New Caledonia, highlighting the emergence of this neglected but highly dangerous disease.

## Materials & methods

### Animals and sampling

**Fig. 1:**
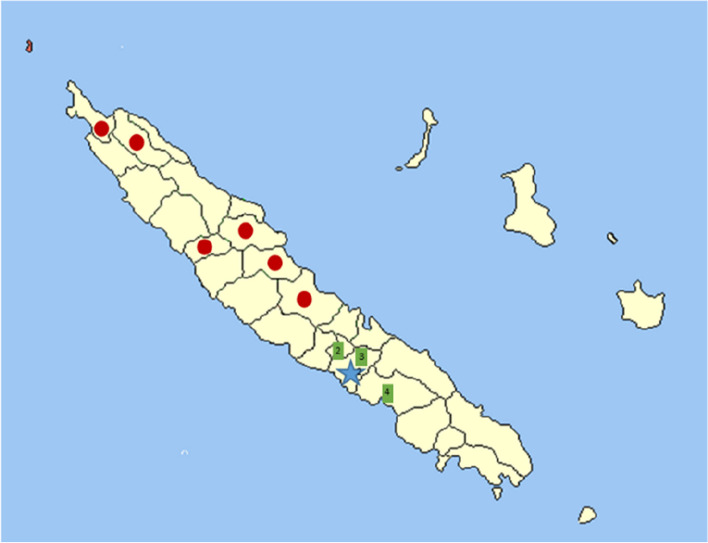
Location of the studied goat focks. The locations of goat flock 1 (blue, with the isolation of a *B. pseudomallei* strain), goat flocks 3 to 4 (green, all negative by serology) and reported human melioidosis cases (red) between 1999 and 2013 [16] in New Caledonia are shown on the map

Four goat flocks located in New Caledonia were included in this study: Flock 1, located on the Lebris peninsula (Foa commune), initially had 18 goats, including the male goat found dead. This male had been part of the flock for a long time and all the goats grazed together in the same area. Blood samples and nasal and/or rectal swabs were collected from these goats in August 2021 and January 2022. Three other goat flocks (Flocks 2 to 4) located in Nessadiou (Bourail commune), Farino, and Camp Brun (Boulouparis commune) were included in the study for further serological investigations, as shown in Fig. 1. Blood samples were collected from ten animals in these flocks in August 2021. All sera and swabs collected were stored at -20°C prior to analysis.

### Bacteriology

Various tissues from the dead male goat, including lung, liver, pleural and testicular abscess samples, were subjected to standard bacteriological examination. They were first cultured on blood agar without specific screening for *B. pseudomallei*. The growing colonies were then identified by MALDI-TOF MS (Microflex, Bruker) using the MBT Compass SR Taxonomy database for identification purposes. After identification, the *B. pseudomallei* strain was grown on Ashdown’s medium for further characterization.

### DNA extraction and PCR analysis

Nasal and rectal swabs were collected and DNA was extracted using the Roche High Pure PCR Template Preparation kit (Roche, France). To ensure accuracy, a PCR inhibition control (Diagenode, Belgium) was added to each sample and to each control prior to DNA extraction. The extracted DNA was eluted with 200 µL of elution buffer. Two PCR methods were used for detection, including a PCR targeting the *B. pseudomallei* complex [17] and a *B. pseudomallei*-specific real-time PCR targeting the orf11 sequence [18]. In addition, a lysate from the *B. pseudomallei* culture was tested using the same PCR methods described above for species confirmation. The lysate was obtained by boiling the bacterial suspension for 20 min and centrifuging at 13,000 rpm for 10 min.

### Whole genome sequencing and in silico molecular analysis

The *B. pseudomallei* strain was subjected to both short paired-end and long read sequencing after DNA extraction using the MasterPure™ Complete DNA and RNA Purification kit (Lucigen®, France) according to the manufacturer’s instructions. The DNA Prep kit (Illumina San Diego, CA, USA) and the Native Barcoding Genomic DNA kit SQK-LSK109 (ONT, Oxford Nanopore Technologies, Oxford, UK) were used for library preparation. The MiSeq platform with V2 reagent and 250 bp paired-end reads was used for short-read sequencing, while long-read sequencing was performed using the MinION and an R9.4.1 flowcell. Guppy v6.0.0 (ONT) was used to base-call the resulting Fast5 files in HAC mode, which were then trimmed using NanoFilt v2.6 [19] to retain only reads longer than 1000 bp with a quality above Q10. De novo assembly was performed using only long reads using Flye v2.9-b1768 [20] with the parameters –genome-size 7.5m –iteration 1. Short reads were then used to polish the assembly using the POLCA genome polishing software from the MaSuRCA analysis toolkit (https://github.com/alekseyzimin/masurca). BWA v0.7.12–5 [21] and minimap2 v2.17-r941 [22] were used to map short and long reads back to the assembly, respectively. Qualimap v2.2.2-dev [23] was used to generate assembly statistics. The resulting genome assembly has been deposited in the ENA database under the accession number ERZ16299634.

For further analysis of the *B. pseudomallei* strain, the seven MLST genes defined for this species were extracted using the MLST software (https://github.com/tseemann/mlst), which uses components from the PubMLST website (https://pubmlst.org/). The MLST genes of 20 isolates from the Pacific region were extracted from the PubMLST website. The concatenated MLST sequences of each isolate were aligned using ClustalW, and phylogenetic tree was constructed using MEGA software (MEGA X), which applied the maximum likelihood method and Kimura 2-parameter.

A SNP-based phylogeny tree was constructed using PhaME, a tool that identifies the core genome from input datasets (including finished genomes, draft assembly contigs, and/or raw fastq reads). PhaME extracts core SNPs, classifies them as coding or non-coding, and further categorizes them as synonymous or non-synonymous SNPs. It then reconstructs a phylogeny and performs molecular evolutionary analysis [24]. The complete genomes downloaded from the PubMLST website were used to construct the SNP-based phylogeny tree, with PhaME identifying 64,209 core SNP positions out of a total of 343,975 core genome positions. The core genome alignment file, including variant and invariant positions, was used to reconstruct the phylogenetic tree using Geneious Tree Builder (Geneious Prime), which applied the neighbor-joining method and the Tamura-Nei parameter.

### Serology methods

The complement fixation test (CFT) is the reference method for equine glanders. It was performed on sera diluted 1:5, as previously described using the Bioveta antigen (Antigen *Burkholderia* (*Pseudomonas*) *mallei* for BRC, Bioveta, Czech Republic) and the cold method [25].

Two ELISA kits (ID Screen® Glanders indirect ELISA, GLANDA ELISA (Innovative Diagnostics, France)), originally developed for the diagnosis of equine glanders, were used according to the recommendations of the manufacturer. The percentage of positivity obtained for each serum sample is given in Table 1 together with the interpretation based on the criteria provided in the instructions. The results correspond to the percentage of positivity compared to the positive control provided in each ELISA kit and calculated from the optical density (OD) values measured at 450 nm according to the following formula: S/P% = ((OD sample—OD negative control)/(OD positive control—OD negative control))* 100.

**Table 1:** Summary of data for animals from flock 1. The table includes serological (CFT, ELISA, Luminex) and PCR results

			1st sampling (August 2021)									2nd sampling (January 2022)		
			**CFT**	**ID Screen ELISA**	**GLANDA ELISA**	**GroEL**	**Luminex** **Hcp1**	**BPSS1840**	**real-time PCR** (orf11)		**ID Screen ELISA**	**GLANDA ELISA**	**real-time PCR** (orf11)	**Comments**
**Animal Id**	year of birth	sex	(Titer)	(% Positivity) D (40–50%) P (> 50%)	(% Positivity) P (≥ 70%)	% Positivity	% Positivity	% Positivity	nasal swab (Ct)	rectal swab (Ct)	(% Positivity) D (40–50%) P (> 50%)	% Positivity P (≥ 70%)	nasal swab (Ct)	
2088	2018	F	n	D (47%)	P (394%)	57	77	71	n	n	P (63%)	P (464%)	n	mother of de Castille/Casse-tête
Castille	2021	F	n	P (93%)	P (343%)	8	103	1	P (32,9)	n	n (0%)	n (0%)	n	daughter of no. 2088 (sister of Casse-tête)
Casse-Tête	2021	F	D (1)	P (105%)	P (352%)	18	100	88	n	n	n (0%)	n (1%)	n	daughter of no. 2088 (sister of Castille)
2090	2018	F	n	n (1%)	n (0%)	27	2	100	n	n	n (3%)	n (1%)	n	mother of Strange
Strange	2021	M	n	n (1%)	n (1%)	9	1	19	n	n	n (1%)	n (0%)	n	son of no. 2090
2092	2018	F	n	n (5%)	n (0%)	35	4	44	P (36,3)	n	n (2%)	n (2%)	n	mother of no. 2086/2089 and Marvelle
2086	2019	F	D (1)	P (85%)	P (379%)	100^a^	100^a^	100^a^	n	n	P (76%)	P (496%)	n	daughter of no. 2092
2089	2019	F	n	n (7%)	P (336%)	33	74	57	n	n	n (5%)	P (252%)	n	daughter of no. 2092
Marvelle	2021	F	n	n (1%)	n (0%)	14	0	4	n	n	n (1%)	n (1%)	n	daughter of no. 2092
2098	2017	F	n	n (1%)	n (0%)	8	4	12	n	n	n (0%)	n (0%)	n	mother of no. 2087 and Prince/Princesse
2087	2019	F	n	D (41%)	P (369%)	38	104	117	n	n	n (38%)	P (440%)	n	daughter of no. 2098
Prince	2021	M	n	n (1%)	n (0%)	7	0	11	n	n	n (1%)	n (2%)	n	son of no. 2098 (brother of Princesse)
Princesse	2021	F	n	n (0%)	n (1%)	9	0	3	n	n	n (0%)	n (0%)	n	daughter of no. 2098 (sister of Prince)
Martine^a^		F												
2094	2019	F	D (2)	n (2%)	n (2%)	10	2	97	n	n	n (5%)	n (2%)	n	daughter of no. Martine (dead 2 days before of the male goat)
Martin	2021	M	n	n (-1%)	n (0%)	14	0	3	n	n	n (2%)	n (1%)	n	son of no. Martine (dead 2 days before of the male goat)
2099	2017	F	n	n (3%)	n (1%)	11	1	81	n	n	n (3%)	n (2%)	n	
Jesus	nd	M	n	n (2%)	n (0%)	10	1	27	n	n	/	/	/	
Taxi^a^	2018	M												

^a^: dead

The latex immunoagglutination (LIA) test, developed at the Emerging Pathogens Institute, University of Florida [26], is suitable for both human and animal melioidosis diagnosis. The test was only performed on the serum from animal no. 2086, collected in August 2021 from flock 1, according to the recommended test procedure. The test included five antigens (AhpC (BPSS0492), a common LPS antigen type A from *B. pseudomallei* and *B. thailandensis,* a rare LPS antigen from *B. pseudomallei* serotype B, Manno-heptose CPS, and Hcp1). Agglutination with at least two of these antigens is required to declare the specimen positive.

The protein array test used in this study was developed at the University of Graz and consists of 21 recombinant *B. pseudomallei* proteins [13]. Originally developed for human diagnosis, this test has recently been evaluated for equine glanders [27]. In our study, the test was specifically adapted for goats, using the serum of the ELISA positive animal no. 2086 of flock 1, collected in August 2021, and a serum sample from a healthy French goat tested under different conditions: diluted 1:1000 and 1:2000 and tested with the anti-goat biotin-SP (long spacer) AffiniPure rabbit anti-goat IgG, Fc fragment specific (Jackson, USA) conjugate diluted 1:1000 or 1:2500. The difference in OD_450nm_ (delta OD_450nm_) between the negative and the serum sample no. 2086 was calculated for each sample and conjugate dilution.

The Luminex assay, originally developed for the diagnosis of equine glanders [28], was adapted for the analysis of goat sera using the conjugate Biotin-SP (long spacer) AffiniPure Rabbit Anti-Goat IgG, Fc fragment specific (Jackson, USA) diluted at 1:5000. Serum from animal no. 2086 of flock 1, collected in August 2021, was selected as a positive control to determine the S/P value calculated for each antigen. The assay was performed with GroEL (BPSL2697), Hcp1 (BPSS1498) and BPSS1840 antigens as previously published [27, 28].

## Results

### Isolation of a B. pseudomallei strain from abscesses of a male goat

**Fig. 2:**
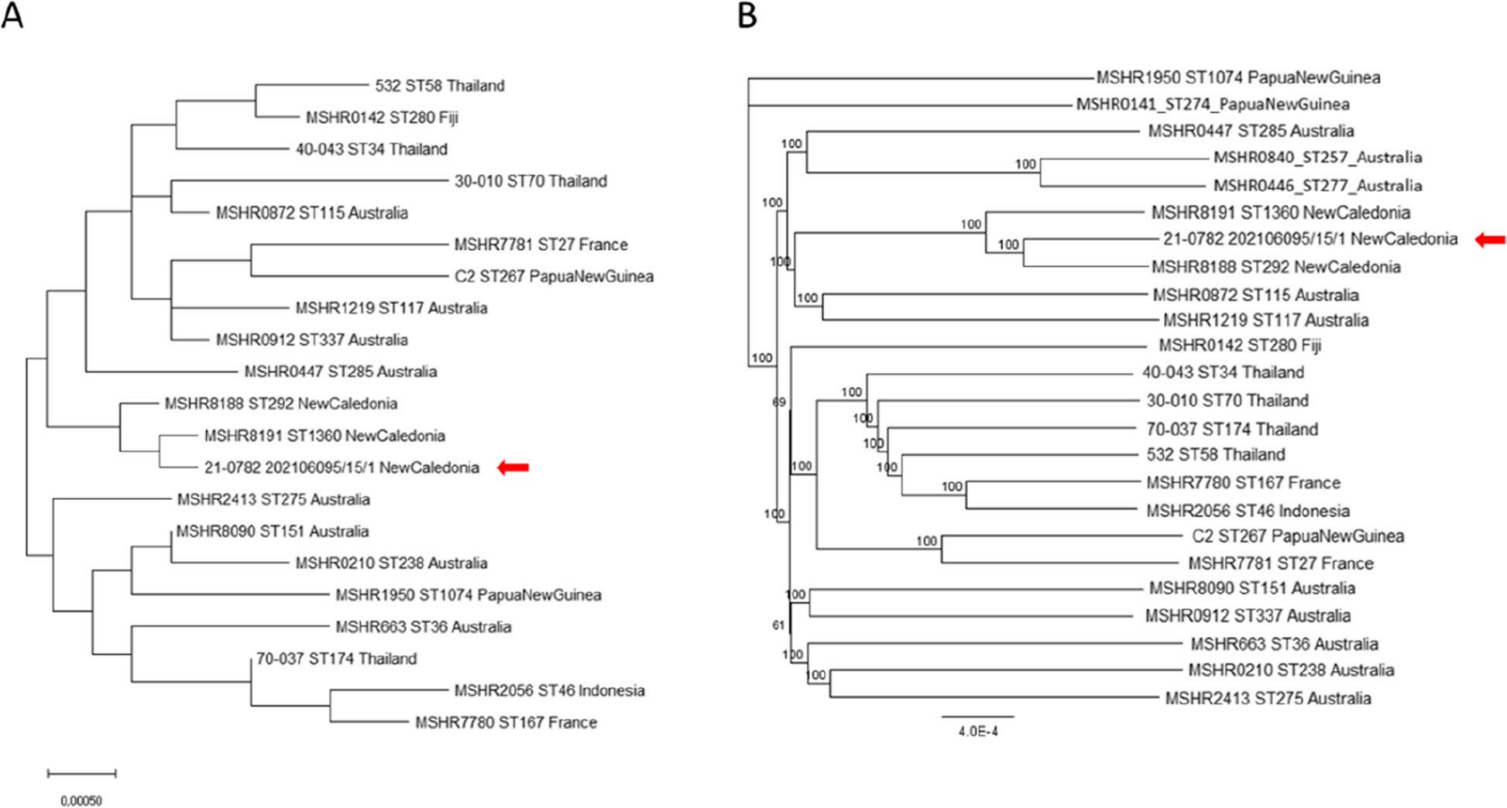
MLST and SNP-based *B. pseudomallei* phylogeny. Phylogeny of a selection of *B. pseudomallei* strains based on their Multi-Locus Sequence Typing (MLST) (**A**) (Maximum Likelihood method and Kimura 2-parameter model in the MEGA X software) and SNP (**B**) (Neighbor-Joining method and Tamura-Nei parameter in the Geneious Prime software) profiles. The strain isolated from a goat in this study (21-0782_202106095/15/1) is indicated by an arrow

In June 2021, a pure culture of *B. pseudomallei* was obtained from the pleural and testicular abscesses of a goat found dead, while *E. coli* was isolated from its liver and lungs. *B. pseudomallei* was identified by MALDI-TOF MS and confirmed by PCR and whole genome sequencing. The 7 MLST genes of this strain were extracted from its genomic sequence and correspond to MLST allelic sequences already deposited in the PubMLST database (*ace*(9), *glt*B(2), *gmh*D(3), *lep*A(4), *lip*A(6), *nar*K(8), *ndh*(1)), but whose combination corresponds to a new ST profile (ST2045) that clusters with those of the human strains described so far in New Caledonia (Fig. 2A). Furthermore, the analysis of SNPs extracted from the genome of this strain and those circulating in the Australia/New Caledonia region, confirms its clustering with the local strains (Fig. 2B).

### Further testing of the flock and other neighboring flocks

#### First screening by CFT, ELISA and PCR analysis

Following the isolation of a *B. pseudomallei* strain, additional testing of the remaining animals on the flock was conducted in August 2021, as samples from the dead goat were no longer available. Nasal and rectal swabs were collected from the 17 remaining goats (flock 1) and tested by PCR. Two nasal swabs were slightly positive for *B. pseudomallei* (Castille and animal no. 2092, with Ct values of 32.9 and 36.3, respectively), confirming the exposure of this flock to this bacterium.

Although there are currently no validated serological tools for the diagnosis of melioidosis in goats, this study used multiple methods to test sera from flock 1 and from 3 neighboring goat flocks that have never reported cases of melioidosis.

In particular, serological tests developed for the diagnosis of glanders were used, with technical adaptations (e.g. anti-goat conjugate) where necessary. The complement fixation test was used, as well as two ELISA kits whose detection systems allowed the detection of goat antibodies (either with a multi-species conjugate for the ID Screen® Glanders indirect ELISA kit or with a double antigen system that does not require a conjugate for the GLANDA ELISA kit). The results obtained in flock 1, where a strain of *B. pseudomallei* was isolated, are summarised in Table 1.

The complement fixation test gave only weak reactions (maximum 50% inhibition at 1/5 dilution) and none of the sera was found to be positive by this method according to the criteria used for the diagnosis of equine glanders. With the ELISA kits, 3 positive results (Castille, Casse-tête, no. 2086) and 2 doubtful results (nos. 2088 and 2087) were obtained with the ID Screen® Glanders indirect ELISA kit (based on a semi-purified suspension of *B. mallei*) and 6 positive results (Castille, Casse-tête, nos. 2086, 2087, 2088, and 2089) were obtained with the GLANDA ELISA kit (based on a recombinant *B. mallei* protein). Five of these positive samples were also positive or doubtful with the ID Screen® Glanders indirect ELISA kit. It should be noted that the percentage of positivity for the samples identified as negative was at most 7% for the ID Screen® Glanders indirect ELISA kit and 2% for the GLANDA ELISA kit, whereas the percentage of positivity for the samples identified as positive showed a mean value of 94% and 362%, respectively (Table 1). None of the sera collected from three other goat flocks (flocks 2 to 4) in the same geographical area tested positive by CFT and the two ELISA tests (Supplementary Table 1).

#### Follow-up ELISA and PCR analysis in flock 1

Goats sampled in flock 1 in August 2021 were re-analysed in January 2022. Of the six animals that initially tested positive with the GLANDA ELISA kit, only four (nos. 2086, 2087, 2088, and 2089) remained positive with this kit. When tested with the ID Screen® Glanders indirect ELISA kit, of the 5 animals that initially tested positive or doubtful with this kit, only animals nos. 2088 and 2086 were found to be positive. Notably, animal no. 2088 had a doubtful result with this kit at the first sampling time (as shown in Table 1).

At the second sampling time, all nasal swabs were negative for *B. pseudomallei* by PCR.

#### Detailed analysis of recognized antigens in serum sample no. 2086 from flock 1 using LIA and array assays

Sample no. 2086 from flock 1 was found to be positive with both ELISA tests for both sampling times. This sample was therefore selected for further analysis. First, the serum sample was tested by LIA, which showed positive signals with the common LPS antigen and with Hcp1 (as shown in Supplementary Fig. 1).

**Fig. 3:**
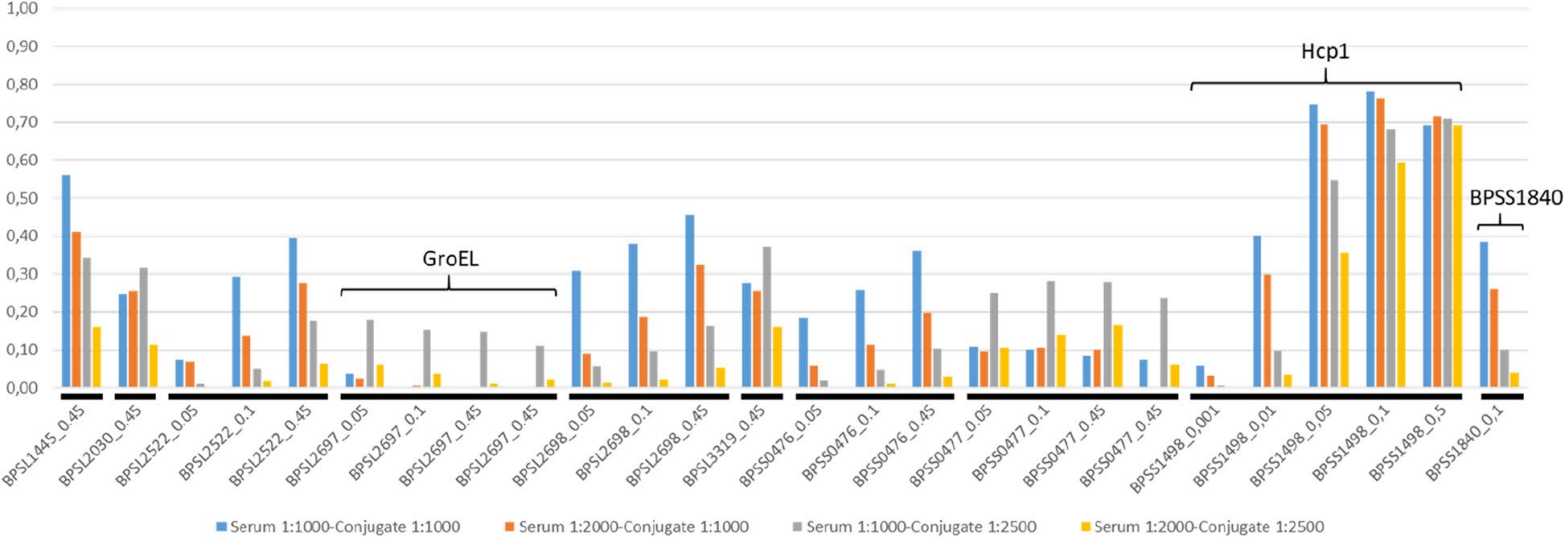
Analysis on the array assay of the serum sample no. 2086 from flock 1. This figure shows the delta OD_450nm_ values for 10 antigens (some plotted at different concentrations (mg/mL)) showing the difference between sample no. 2086 from flock 1 and a French goat serum sample (used as a negative control). The tests were conducted at two dilutions (1:1000 and 1:2000) and with two conjugate dilutions (1:1000 and 1:2500). BPSL2697 corresponds to GroEL, BPSS1498 corresponds to Hcp1

The sample was then analyzed using an array assay and results showed differences in 10 out of 21 antigens between the negative sample and the serum sample from animal no. 2086. These antigens include: BPSL1445 putative lipoprotein, BPSL2030 putative exported protein, BPSL2522 (OmpA), BPSL2697 (GroEL), BPSL2698 (GroES), BPSL3319 (Flagellin FliC), BPSS0476 (GroES), BPSS0477 (GroEL), BPSS1498 (Hcp1), and BPSS1840 (putative N-acetylmuramoyl-L-alanine amidase) (as shown in Supplementary Table 2 and Fig. 3).

#### Evaluation of the Hcp1, GroEL and BPSS1840 by Luminex, as serological markers of exposure to B. pseudomallei in 4 different flocks, including flock 1

**Fig. 4:**
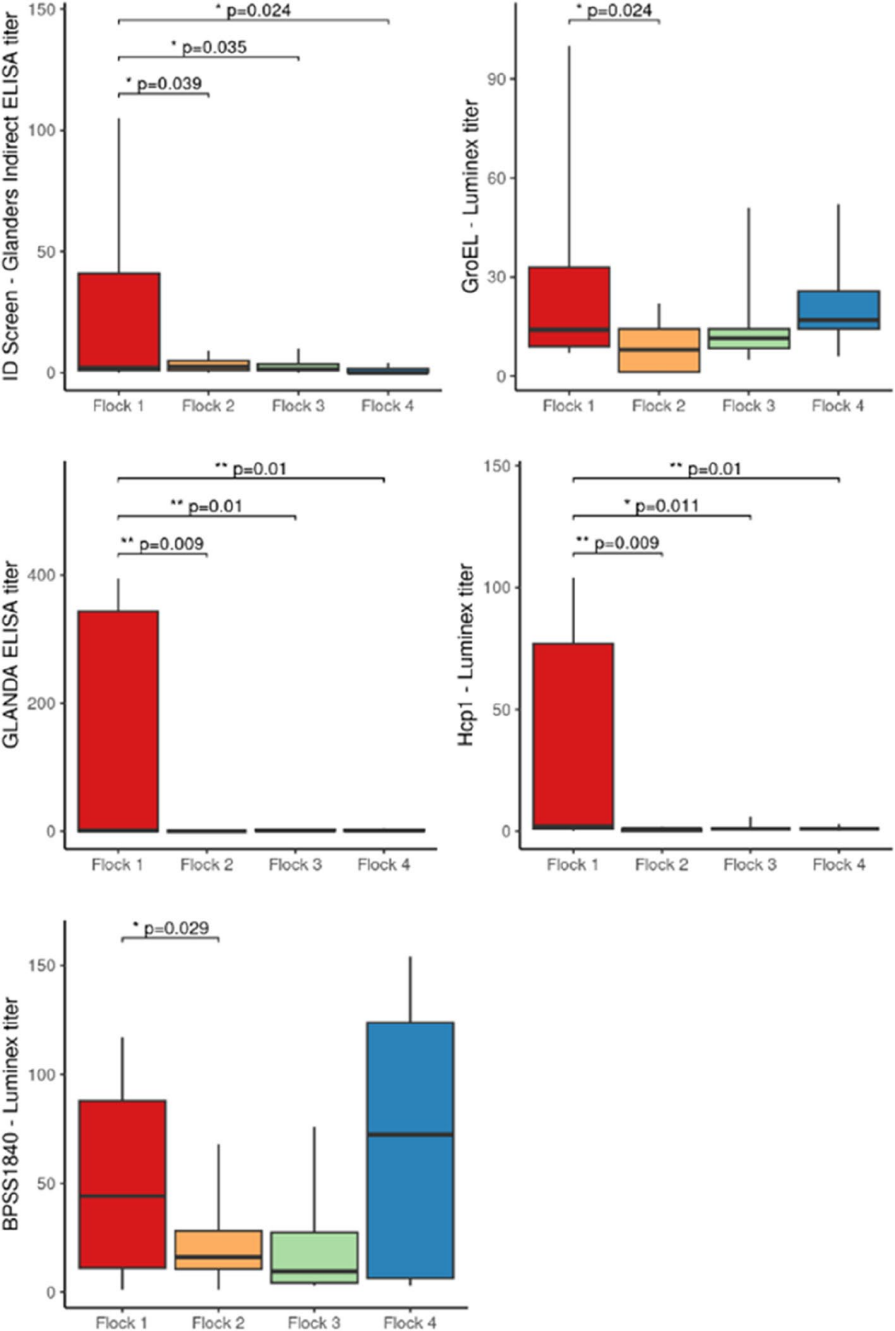
Comparison of the different serological tests in the 4 goat flocks. This graph shows the comparison of the mean test values for each flock (calculated as the mean of the values obtained for all animals in a flock), using different methods: ID Screen® Glanders indirect ELISA kit, GLANDA ELISA kit and Luminex (targeting GroEL, Hcp1, and BPSS1840 proteins)

Based on these results, sera collected in August 2022 from flocks 1 to 4 were tested by Luminex. The method used was originally developed for glanders [28], and was expanded to include the BPSS1840 protein and adapted for goat diagnosis. Indeed, an anti-goat conjugate and the serum from goat no. 2086 in flock 1 (as positive control for the assay) were used. The three proteins (GroEL, Hcp1, and BPSS1840) used in the Luminex assay have been previously identified in other studies as being of interest in the diagnosis of clinical glanders cases [27, 28]. Sera collected from goats on farms with (flock 1) or without (flocks 2 to 4) reported cases of melioidosis were tested and the results are presented in Table 1 and Fig. 4.

Although there are no established interpretation criteria for these antigens for melioidosis, a percentage of positivity was calculated for each serum sample using the serum from goat no. 2086 in flock 1 as a positive control for the calculation formula. Student’s T-test showed significant differences between flock 1 and the three other flocks for the titers observed using Hcp1 with Luminex, the GLANDA ELISA kit and the ID Screen® Glanders indirect ELISA kit. Significant differences were also observed for GroEL and BPSS1840 Luminex titers, but only between flock 1 and flock 2.

## Discussion

Following the detection of *B. pseudomallei* on a goat farm in New Caledonia, the remaining animals were tested. Despite regular reports of human melioidosis cases since 1999 [15, 16], this is the first animal case confirmed in New Caledonia, although a 1984 study identified seropositive animals in several species [16]. The analysis of the *B. pseudomallei* strain isolated from the abscesses of a male goat led to the identification of a novel MLST sequence type (ST2045) characterized by a unique combination of previously identified alleles. This sequence type is related to those identified in human melioidosis cases in the region (ST292 and ST1360) [16], suggesting geographic specificity, a finding supported by our phylogenetic analysis using single nucleotide polymorphisms.

Prior to the death of the male goat, another female had succumbed to severe coughing 2 days earlier, although most of the animals typically recovered from such episodes. Bacteriological analysis of tissues from the dead male goat revealed *B. pseudomallei* in pleural and testicular abscesses and *E. coli* in the lungs. Both acute and chronic forms of melioidosis have been documented in goats, with clinical signs including fever, weight loss, nasal discharge, cough, severe mastitis, abortion, and orchitis with testicular nodules [14]. Some of these clinical signs have been observed in this flock, but had not been investigated until the analysis of this dead male.

To assess the infection status of the remaining goats in this flock, and given the limited availability of tools to diagnose melioidosis in animals, especially in goats [29, 30], several serological tests were used in our study, including those for glanders [25, 28, 31, 32], which is caused by *B. mallei*, a bacterium closely related to *B. pseudomallei*. Although the complement fixation test has previously been effective in diagnosing melioidosis in goats and pigs [33, 34], the use of this test with a *B. mallei* antigen in our study did not yield positive results, possibly due to the antigen used or the fact that the rest of the animals did not show acute infection. However, we observed positive ELISA results in 4 to 6 animals using two different kits, with the same animals testing positive on both kits. The ID Screen® indirect Glanders ELISA is based on a semi-purified fraction of *B. mallei* [31] and the GLANDA ELISA detects antibodies to an undisclosed recombinant *B. mallei* protein [32]. Five months after the initial testing, follow-up showed that 2 to 4 animals still had high S/P% values, depending on the ELISA test used. In the meantime, the animals were confined to prevent further exposure to *B. pseudomallei*, a measure that likely prevented additional exposure or contamination of the animals. On this farm, infrequent but persistent episodes of heavy rainfall likely facilitated the persistence of *B. pseudomallei* due to the clayey nature of the soil [1] and the exposure of the animals. Interestingly, three nearby farms with no reported cases of melioidosis had negative results in both ELISA tests, indicating very low S/P% values. These S/P% values clearly discriminate between positive and negative animals within flock 1 and when compared to neighboring herds, indicating the potential effectiveness of these ELISA kits in detecting *B. pseudomallei* exposure in goats. However, further research is needed to determine whether the interpretation criteria established for the diagnosis of glanders need to be adjusted.

Recombinant proteins are of interest for enhancing the specificity of disease diagnosis, as illustrated for human melioidosis [12] and glanders [27, 31]. However, proteins require individual evaluation to determine their value in both clinical and chronic contexts. Sera from the remaining goats in flock 1 and sera from the 3 neigbouring farms were tested for three antigens of interest in the diagnosis of human melioidosis and/or glanders (Hcp1, GroEL and BPSS1840) [12, 35, 36]. The results showed no detectable response to GroEL in any of the flocks, and responses to Hcp1 were only observed in flock 1, correlating with results from the GLANDA ELISA kit. Of note, responses to BPSS1840 were observed in flocks 1 and 4, although there were no reported cases of melioidosis in flock 4. This suggests that animals in this flock may have been exposed. Further analysis of environmental samples in the vicinity of these flocks is required to confirm the presence of *B. pseudomallei* and to assess the potential exposure of these animals. Interestingly, there was no response in flock 1 to GroEL, a marker of interest in the clinical diagnosis of glanders. In flock 1, the seropositive animals had no lesions or clinical signs, but may be either in the early stages of infection or simply exposed to *B. pseudomallei* or another environmental *Burkholderia,* possibly causing immunological cross-reactions [37]. Analysis of the serum from the dead goat for its response to these three antigens would have been beneficial in the event of a confirmed infection, but unfortunately this sample was not available.

In parallel, we analysed an ELISA-positive serum from flock 1 against twenty proteins coated on the array and identified ten potential candidate proteins. Further analysis is required to determine the relevance of these proteins in the diagnosis of caprine melioidosis. The Hcp1 protein emerged as significant in our analyses, identified by Luminex, LFI and the array test. The BPSS1840 protein, identified in herd 1 by both the Luminex and array tests, and in herd 4 by Luminex, the only test performed in that herd, requires further analysis. In addition, among the top 10 antigens of interest in our array analysis, the OmpA or FliC antigens have recently shown promise for diagnosis of melioidosis in goats [30].

The isolation of a strain of *B. pseudomallei* prompted a review of the farm's management practices and potential exposures. As a result, the following recommendations were made: 1) Isolate seropositive and/or PCR-positive animals in a building or at least in a paddock that is not flooded. 2) Prevent animals from continuous access to potentially contaminated areas. 3) Chlorinate drinking water where possible. Keep in mind that animals may still be drinking from hilltop reservoirs, false bogs, and standing water. 4) Wear PPE, including at least gloves and a mask, and disposable clothing for sampling and necropsy when caring for sick animals (veterinarians and breeders). 5) Require a clinical examination of all animals leaving the farm (on the hoof or for slaughter) to ensure that sick animals (those with respiratory problems or abscesses) are not sent to the slaughterhouse or to another farm. 6) Euthanize sick animals (those with fever, respiratory problems, abscesses, arthritis, mastitis, etc.) and incinerate dead animals.

This study confirms the presence of *B. pseudomallei* in New Caledonia, the importance of One Health approaches, and highlights the need for increased awareness among all animal and human health partners, including farmers, veterinarians, and biologists.

In summary, after the first isolation of a *B. pseudomallei* strain from a goat abscess in New Caledonia, a survey of the livestock was initiated. The survey revealed that several animals were seropositive, suggesting that they had been exposed to the bacteria or a closely related species causing cross-reactivity. Serological analyses were performed using ELISA kits developed for the diagnosis of equine glanders, as few serological tests are currently available for the diagnosis of melioidosis in animals, particularly in goats. Further studies are needed to develop more specific and sensitive serological tests for the diagnosis of melioidosis in animals and to better understand the epidemiology of the disease in New Caledonia.

### Supplementary Information

**Supplementary Material 1.**

**Supplementary Material 2.**

**Supplementary Material 3.**

## Data Availability

All data generated or analyzed during this study are included in this published article. The data sets generated during and/or analysed during the current study are available from the corresponding author.
