# Supplementary material for: Isolation of Burkholderia pseudomallei from a goat in New Caledonia: implications for animal and human health monitoring and serological tool comparison
Source: BMC Vet Res. 2024 Mar 23;20:114. doi: 10.1186/s12917-024-03957-5 (PMC10960402; doi:10.1186/s12917-024-03957-5)
Supplement: Supplementary file 1 — Supplementary Material 1. [file 12917_2024_3957_MOESM1_ESM.pptx]

## Slide 1
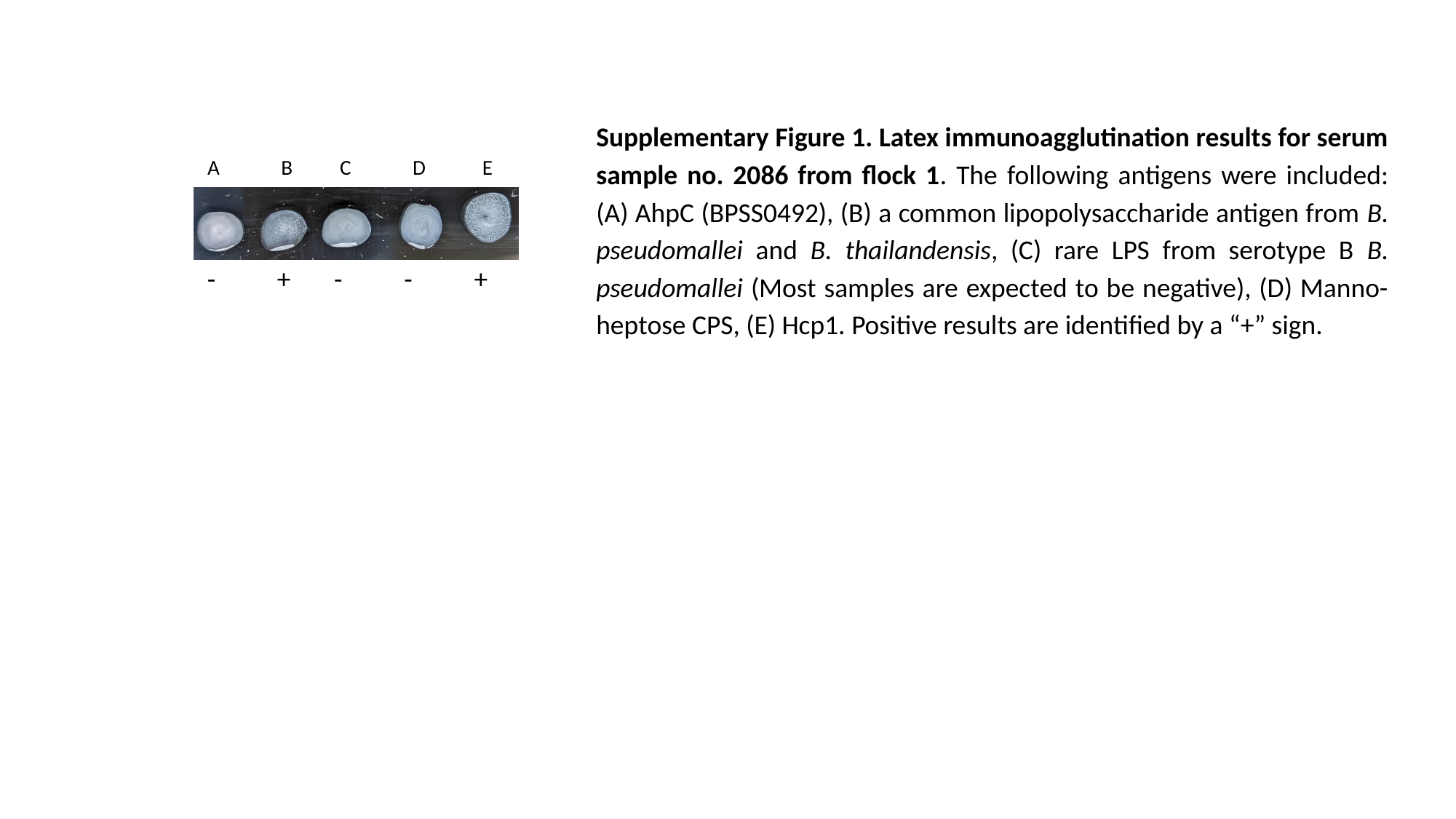

Supplementary Figure 1. Latex immunoagglutination results for serum sample no. 2086 from flock 1. The following antigens were included: (A) AhpC (BPSS0492), (B) a common lipopolysaccharide antigen from B. pseudomallei and B. thailandensis, (C) rare LPS from serotype B B. pseudomallei (Most samples are expected to be negative), (D) Manno-heptose CPS, (E) Hcp1. Positive results are identified by a “+” sign.
A B C D E
- + - - +
